# MiR-26b is down-regulated in carcinoma-associated fibroblasts from ER-positive breast cancers leading to enhanced cell migration and invasion

**DOI:** 10.1002/path.4248

**Published:** 2013-10-09

**Authors:** Eldo T Verghese, Ruth Drury, Caroline A Green, Deborah L Holliday, Xiaomei Lu, Claire Nash, Valerie Speirs, James L Thorne, Helene H Thygesen, Alexandre Zougman, Mark A Hull, Andrew M Hanby, Thomas A Hughes

**Affiliations:** 1 Leeds Institute of Molecular Medicine, University of LeedsLeeds, UK; 2 Department of Histopathology, St James's University HospitalLeeds, UK; 3 Statistics and Bioinformatics, Leeds Cancer Research UK Centre, St James's University HospitalLeeds, UK

**Keywords:** fibroblast, stroma, microRNA, tumour microenvironment, microRNA-26b

## Abstract

Carcinoma-associated fibroblasts (CAFs) influence the behaviour of cancer cells but the roles of microRNAs in this interaction are unknown. We report microRNAs that are differentially expressed between breast normal fibroblasts and CAFs of oestrogen receptor-positive cancers, and explore the influences of one of these, miR-26b, on breast cancer biology. We identified differentially expressed microRNAs by expression profiling of clinical samples and a tissue culture model: miR-26b was the most highly deregulated microRNA. Using qPCR, miR-26b was confirmed as down-regulated in fibroblasts from 15 of 18 further breast cancers. Next, we examined whether manipulation of miR-26b expression changed breast fibroblast behaviour. Reduced miR-26b expression caused fibroblast migration and invasion to increase by up to three-fold in scratch-closure and trans-well assays. Furthermore, in co-culture with MCF7 breast cancer epithelial cells, fibroblasts with reduced miR-26b expression enhanced both MCF7 migration in trans-well assays and MCF7 invasion from three-dimensional spheroids by up to five-fold. Mass spectrometry was used to identify expression changes associated with the reduction of miR-26b expression in fibroblasts. Pathway analyses of differentially expressed proteins revealed that glycolysis/TCA cycle and cytoskeletal regulation by Rho GTPases are downstream of miR-26b. In addition, three novel miR-26b targets were identified (*TNKS1BP1*, *CPSF7*, *COL12A1*) and the expression of each in cancer stroma was shown to be significantly associated with breast cancer recurrence. MiR-26b in breast CAFs is a potent regulator of cancer behaviour in oestrogen receptor-positive cancers, and we have identified key genes and molecular pathways that act downstream of miR-26b in CAFs. © 2013 The Authors. Journal of Pathology published by John Wiley & Sons Ltd on behalf of Pathological Society of Great Britain and Ireland.

## Introduction

Breast cancer tissue consists of malignant epithelial cells and various other cell types collectively known as tumour stroma 1. It is well established that tumour stroma plays critical roles in controlling breast cancer epithelial cell behaviour, and therefore in defining cancer outcomes 2,3. Tumour stroma includes fibroblasts, inflammatory cells, adipocytes, and blood and lymph vessels 1, but the fibroblasts are typically most abundant and have consequently attracted most attention 4. Gene expression profiles of these carcinoma-associated fibroblasts (CAFs) differ substantially from their normal tissue counterparts 5, although considerable heterogeneity is evident within CAF populations 6,7. Studies have identified specific molecules, such as transforming growth factor β, hepatocyte growth factor 8, stromal cell-derived factor-1 3,6 and phosphatase and tensin homologue 9, that are expressed by CAFs and regulate carcinoma cell behaviour, typically leading to enhanced tumourigenicity. Studies have also identified gene regulatory events that are responsible for the deregulation of such molecules in CAFs, including changes in promoter methylation 10, the activity of key transcription factors 11, and even—controversially—somatic mutations in critical signalling molecules 12. However, the contributions of microRNAs (miRNAs) to gene deregulation in CAFs are virtually unknown.

MiRNAs are a class of more than 1000 non-coding RNAs that regulate the expression of up to 60% of genes 13. They act by binding to mRNAs, usually within 3′ untranslated regions, causing post-transcriptional down-regulation of protein expression by translational repression and/or mRNA destabilization 14. Many studies have demonstrated deregulation of specific miRNAs in breast cancer and the potential functional consequences within epithelial cancer cells 15. Thus, specific miRNAs can themselves be regarded as ‘tumour suppressors’ or ‘oncogenes’. Examples include miR-10a 16, miR-373 17, and miR-21 18, which act as regulators of growth, invasion, and metastasis. Interestingly, there is some evidence that miR-21, which is up-regulated in breast cancers and was assumed to be functional within epithelial cells, is in fact predominantly expressed in fibroblasts 19. Very recently, attention has been given to miRNA roles in the fibroblasts of epithelial cancers, with the first report profiling miRNA expression differences between CAFs and normal fibroblasts (NFs) 20. We examined these differences in great detail and investigated the functional impacts on CAFs of the most consistently deregulated miRNA that we identified, miR-26b.

## Materials and methods

### Ethics, tissue, and laser micro-dissection (LMD)

Ethical approval was obtained (Leeds East REC 06/Q1206/180). LMD was performed using a Zeiss/PALM Microscope (Oberkochen, Germany) from formalin-fixed/paraffin-embedded tissue as described in the Supplementary materials and methods. CAFs were defined as fibroblasts present within tumour masses (as in refs 21 and 22), and less than 2 mm from tumour cells, while NFs were defined by their association with normal epithelium more than 1 cm outside tumour masses. Areas selected were devoid of visible cells other than target cell types. SMA staining [mouse monoclonal; M851 (Dako, Glostrup, Denmark), 1 : 1000] was performed using an IntelliPATH automated stainer (Menarini, Florence, Italy) and the manufacturer's standard conditions.

### Tissue culture, transfection, transduction, and functional assays

MCF7 and HB2 cells were obtained from the European Collection of Cell Cultures. Stable GFP expression was conferred by pTH-GFPa 23, a selectable GFP expression vector. MCF7 cells that stably express firefly luciferase were obtained from Cell Biolabs (San Diego, CA, USA; #AKR-234). Primary fibroblasts were isolated from breast surgical samples 24 and immortalized by hTERT retroviral transduction as described previously 25. Reverse transfection of pre-/anti-miR (Ambion, Carlsbad, CA, USA) and/or plasmids was performed using HiPerFect (Qiagen, Hilden, Germany). Stable miR-26b/control ‘knock-down’ was performed using pmiRZIP lentiviral vectors (System Biosciences, Mountain View, USA). Assays for cell viability, cell cycle, apoptosis, migration, and invasion were performed as described previously 16,26. Flow-cytometry and fluorescence-activated cell sorting were performed on LSRII (BD Biosciences, Franklin Lakes, NJ, USA) and MoFlo (Dako) machines, respectively. Spheroid invasion assays were carried out using modifications of a previously described method 27. Spheroids were formalin-fixed/paraffin-embedded, and 5 µm sections stained with haematoxylin/eosin, or treated for immunohistochemistry (rabbit anti-cytokeratin, Ab9377; Abcam, Cambridge, UK; see ref 28) as above. Dual luciferase assays and pmiRGLO reporter were used (Promega, Fitchburg, WI, USA). Details may be found in the Supplementary materials and methods.

### RNA extraction, quantitative PCR (qPCR), and microarray analyses

RecoverAll Total Nucleic Acid Isolation for FFPE (Ambion) was used for RNA extraction from FFPE. MirVana miRNA Isolation (Ambion) and RNeasy (Qiagen) were used for extraction from cell lines of miRNA and mRNA, respectively. qPCR analyses were performed on 7500/7900HT machines in triplicate with Taqman assays (Life Technologies, Carlsbad, CA, USA). Microarray analyses of miRNA expression were performed using human miRNA v2 arrays and system v1.7 reagents (Agilent, Santa Clara, CA, USA). Further details may be found in the Supplementary materials and methods.

### Protein mass spectrometry

Peptides from processed lysates 29 of cell pellets were separated by capillary liquid chromatography (LC) and analysed by tandem mass spectrometry (MS/MS) using the RSLCnano system and LTQ-Orbitrap Velos mass spectrometer (Thermo Scientific, Waltham, MA, USA). The data search against the IPI Human 3.87 database and label-free quantitation (LFQ) were performed using MaxQuant 1.2.2.5 30. Further details may be found in the Supplementary materials and methods.

### Statistical analyses

Statistical analyses were performed using Prism (GraphPad, La Jolla, CA, USA) with two-tailed tests described in the text 31. Mass spectrometry data were analysed using R (OSX v2.15.1); expression levels were log_2_ median-normalized and analysed using *t*-tests, and *p* values converted to false discovery rates using Benjamini–Hochberg methodology 32.

## Results

### MiRNA expression in breast NFs differs from that in CAFs

We compared the miRNA profiles of breast NFs with those of CAFs using two sources of cells: (i) fibroblasts isolated from formalin-fixed, paraffin-embedded (FFPE) tissue using laser micro-dissection (LMD); and (ii) a tissue culture model in which breast fibroblasts were combined with either non-transformed breast epithelial cells or breast cancer epithelial cells.

**Figure 1:**
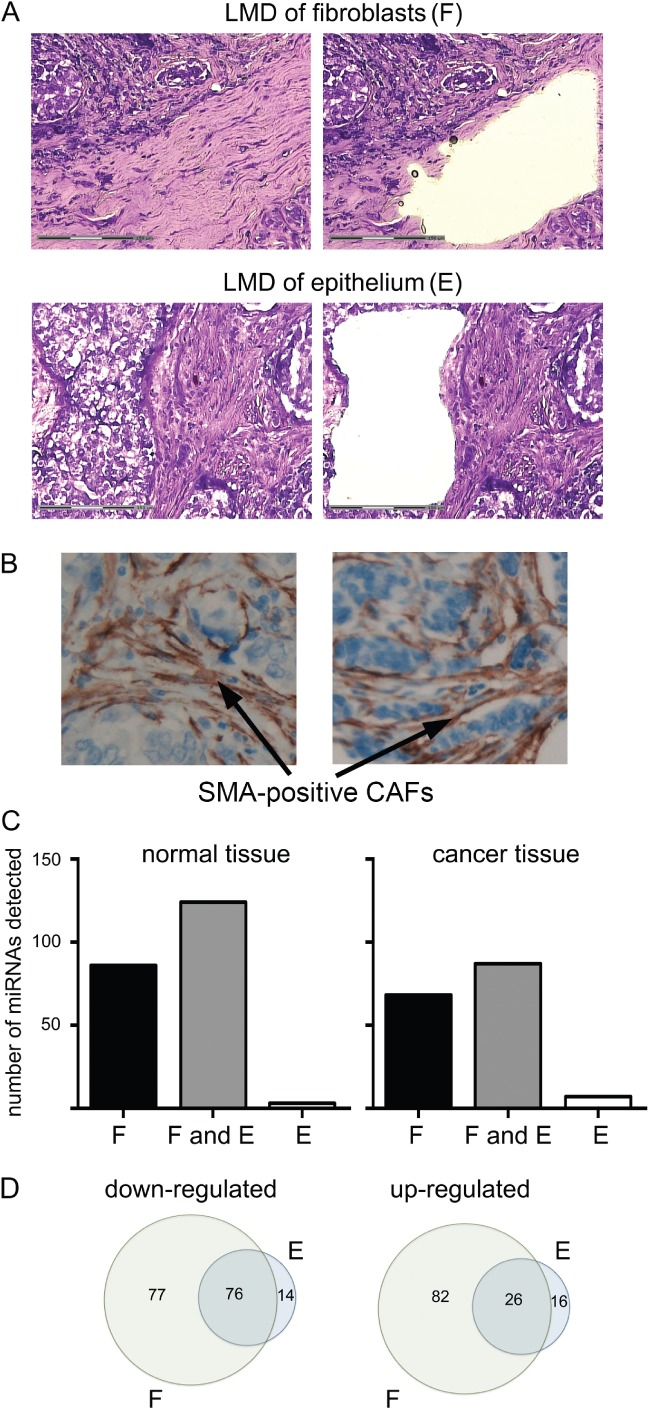
Laser micro-dissection (LMD) allowed analysis of miRNA deregulation in the fibroblast and epithelial cell compartments of breast cancers. (A) Representative images of breast cancer tissue before (left) and after (right) LMD of fibroblast-enriched stroma or epithelial cells as labelled. FFPE breast tissue was sectioned and stained with toluidine blue. Regions for LMD were identified based on morphology. (B) Representative images of tumour sections stained for smooth muscle actin (SMA) using immunohistochemistry. (C, D) Total RNA was extracted from at least 5 mm^2^ of LMD tissue enriched for fibroblasts or epithelial cells from breast cancer tissue or from matched normal breast tissue. Microarray analyses of miRNA expression were performed. (C) Numbers of miRNAs detected in only samples of fibroblast-enriched stroma (F), in both samples of fibroblast-enriched stroma and epithelial cells (F and E), or in only epithelial cell samples (E) are shown for each tissue. (D) Relative expression of each miRNA was compared between normal and cancer tissue within fibroblast-enriched stroma (F) or within epithelial cells (E). Numbers of individual miRNAs that were up- or down-regulated in those compartments are displayed in Venn diagrams showing how many were deregulated in common between compartments (the intersects), or were deregulated in one compartment only.

To validate our LMD protocols, we first prepared samples enriched for either epithelial cells or stromal fibroblasts from tissue blocks of a single breast cancer case [luminal A subtype, (ER+/her2−)] and from matched normal breast tissue. Figure 1A shows representative LMD sections; fibroblast-enriched samples are referred to as fibroblast-enriched stroma (F). Tumour sections were stained for smooth muscle actin (SMA), demonstrating that the majority of CAFs were SMA-positive (Figure 1B). MiRNAs were extracted from LMD samples and microarrays were used to determine expression profiles. Of the 723 miRNAs analysed, 229 (32%) were detected in at least one sample. Many miRNAs were detected in fibroblast-enriched stroma that were not detected in epithelial cells from the same tissue (normal tissue: 86; cancer tissue: 68; Figure 1B), demonstrating that LMD had successfully allowed enrichment for separate cell populations and that the fibroblast-enriched stroma and epithelial miRNA profiles differed. Very few epithelial-specific miRNAs were identified (normal tissue: 3; cancer tissue: 7; Figure 1B). We then examined differential expression between normal and cancer as determined in fibroblast-enriched stroma or epithelial samples (Figure 1C). Seventy-six and 26 miRNAs were found to be down- or up-regulated, respectively, in both epithelium and fibroblast-enriched stroma, indicating some commonality between miRNA deregulation in these breast cancer compartments. A large number of miRNAs were found to be deregulated solely within fibroblast-enriched stroma (77 down; 82 up), while epithelial cell-specific deregulation was relatively rare (14 down; 16 up).

**Figure 2:**
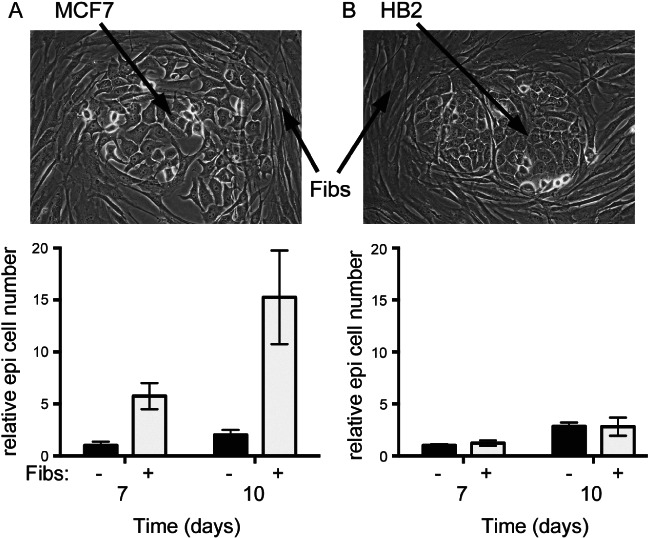
Growth of MCF7 breast cancer epithelial cells, but not non-transformed ‘normal’ HB2 breast epithelial cells, was stimulated by immortalized breast fibroblasts. GFP-labelled MCF7 breast cancer cells (A) or HB2 benign breast epithelial cells (B) were co-cultured with immortalized breast fibroblasts (pictured), or were cultured alone, and epithelial cell growth was monitored by counting GFP-positive cells using flow cytometry for up to 10 days. Data are means of biological triplicates (± standard error) and are representative of duplicate experiments.

MiRNA expression was also examined in a tissue culture model. Immortalized breast fibroblasts were co-cultured with either HB2 cells, breast epithelial cells of non-cancer origin considered to represent normal epithelium, or MCF7 cells, representative of epithelial cells of the most common breast cancer subtype (luminal A). Epithelial cell lines were stably labelled with GFP in order to allow their separate analysis within co-cultures. Fibroblasts had a potent and differential effect on the growth of the two epithelial cell types; the growth of MCF7 cancer cells was stimulated more than five-fold by fibroblasts, while the growth of HB2 cells was not altered (Figure 2). Thus, fibroblasts within the fibroblast/MCF7 co-culture behaved in a manner analogous to CAFs 6, while those within the fibroblast/HB2 co-culture exhibited less functional cross-talk. Fibroblasts were co-cultured with HB2 or MCF7 cells for 9 days before being isolated by fluorescence-activated cell sorting. MiRNA expression within these fibroblasts was examined using microarrays. Two hundred and twenty-six (31%) of the 723 miRNAs analysed were detected in at least one sample. One hundred and sixty-six miRNAs were expressed at lower levels, and 60 miRNAs were more highly expressed in the fibroblasts when they were cultured with MCF7 cancer cells (the CAF model), compared with those cultured with benign HB2 cells (the NF model).

Next, we identified miRNAs that were consistently differentially expressed both between NFs and CAFs from clinical samples and between the tissue culture model ‘NFs’ and ‘CAFs’ (complete dataset—Supplementary Data 1). One hundred and four miRNAs were down-regulated and ten miRNAs were up-regulated in CAFs in both assays (see Supplementary Data 2), representing a remarkably high degree of overlap (eg 69% of LMD-identified down-regulations were also seen in tissue culture, while 63% of tissue culture-identified down-regulations were also seen using LMD). Candidate miRNAs were further filtered using a cut-off of fold changes greater than 10 in both assays, which left six miRNAs that showed consistent and substantial down-regulation (Table1).

**Table 1:** Six miRNAs are consistently down-regulated more than ten-fold in breast CAFs compared with breast NFs in both tissues (comparing matched NF-enriched stroma and CAF-enriched stroma prepared by LMD; Figure 1) and a co-culture model [comparing immortalized breast fibroblasts co-cultured either with the benign breast epithelial cell line HB2 (representing NFs) or with the breast carcinoma cell line MCF7 (representing CAFs); Figure 2]. ‘GeoMean fold change’ is the Geomean of the fold change in tissues and in the co-culture model

MiRNA	Regulation	Fold change (tissues)	Fold change (co-culture)	GeoMean fold change
MiR-7f	Down	47.6	10.2	22
Let-7 g	Down	15	11.4	13
MiR-107	Down	13.1	11.5	12.3
MiR-15b	Down	14.2	10.8	12.4
MiR-26b	Down	21	49.3	32.2
MiR-30b	Down	12.3	30.2	19.3

### MiR-26b is down-regulated in breast cancer CAFs

**Figure 3:**
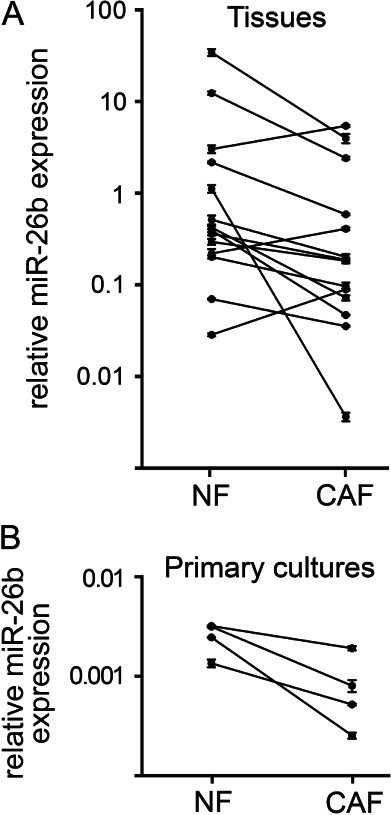
MiR-26b expression was frequently down-regulated in CAFs compared with matched NFs. (A) Samples enriched for fibroblasts were isolated by LMD from samples of matched breast cancer and normal tissue from 14 sequential cases of luminal A breast cancer (see Supplementary Table 1). (B) Primary cultures of matched NFs and CAFs were established from four further breast cancer cases. qPCR was used to analyse miR-26b expression relative to the geometric mean RNU6B and RNU48. Data are means of technical triplicates (± standard error).

MiR-26b was examined further as it showed the greatest mean fold change (Table1). Further samples enriched for NFs or CAFs were prepared by LMD from archival tissue blocks representing an additional 14 sequential cases of luminal A subtype breast cancers (defined as ER+/her2−). MiR-26b expression was quantified using qPCR (Figure 3A). MiR-26b was down-regulated in CAF-enriched stroma compared with NF-enriched stroma in 11/14 cases (Figure 3A; Wilcoxon matched-pair signed rank *p =* 0.04). We also examined miR-26b expression in matched primary cultures of NFs and CAFs isolated from four more breast cancer cases; miR-26b was down-regulated in CAFs in all cases (Figure 3B). Therefore, we found miR-26b to be down-regulated in CAFs in 15/18 cases in our validation cohort. Supplementary Table 1 shows the clinical features of this cohort.

### Reduced miR-26b activity in fibroblasts inhibits growth but enhances migration and invasion

**Figure 4:**
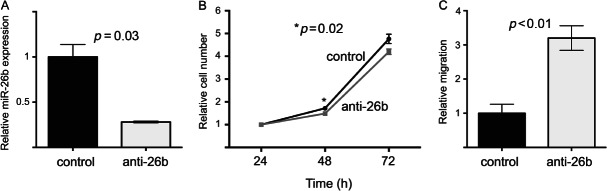
Transient miR-26b down-regulation in breast fibroblasts inhibited growth but stimulated migration. Immortalized breast fibroblasts were transiently transfected with anti-miR-26b molecules or with control anti-miRs. (A) MiR-26b expression was quantified 24 h after transfection using qPCR (relative to RNU6B). Data are means of technical triplicates (± standard error) and are representative of duplicate experiments. (B) Cell growth was monitored by MTT assays over 72 h. (C) Migration of fibroblasts was assessed 24 h after transfection using trans-well migration assays by manual counting of cells that had passed through the membranes. Data in B and C are means of biological triplicates (± standard error) and are representative of duplicate experiments.

We examined whether manipulation of miR-26b levels resulted in changes in fibroblast behaviour. Firstly, we transiently transfected immortalized breast fibroblasts with anti-miR-26b or control molecules. We used qPCR to assess the degrees of miR-26b knock-down in these transfections. MiR-26b expression was reduced by more than three-fold (Figure 4A; *p =* 0.03), although it is worth noting that this may under-represent the reduction in miR-26b function since some miR-26b molecules detected may have been functionally sequestered in the cells by anti-miR-26b molecules. Transient knock-down of miR-26b caused a small but statistically significant reduction in fibroblast growth (Figure 4B; *p =* 0.02 at 48 h). We also measured the migration of anti-miR-26b or control transfected fibroblasts using trans-well assays. Migration was significantly increased by more than three-fold by anti-miR-26b transfection (Figure 4C; *p =* 0.008). We also investigated the consequences of miR-26b overexpression by transiently transfecting fibroblasts with pre-miR-26b or control molecules. MiR-26b overexpression caused dramatic and rapid cell death (Supplementary Figure 1), as has been observed previously in colorectal cancer cells 33. However, qPCR analyses revealed that miR-26b was overexpressed by more than 1000-fold in these transfections (Supplementary Figure 1C). We believe that this lacks clinical relevance since this degree of overexpression greatly exceeded differential miR-26b expression in matched NFs and CAFs (Figure 3); therefore, we did not proceed with further overexpression studies.

**Figure 5:**
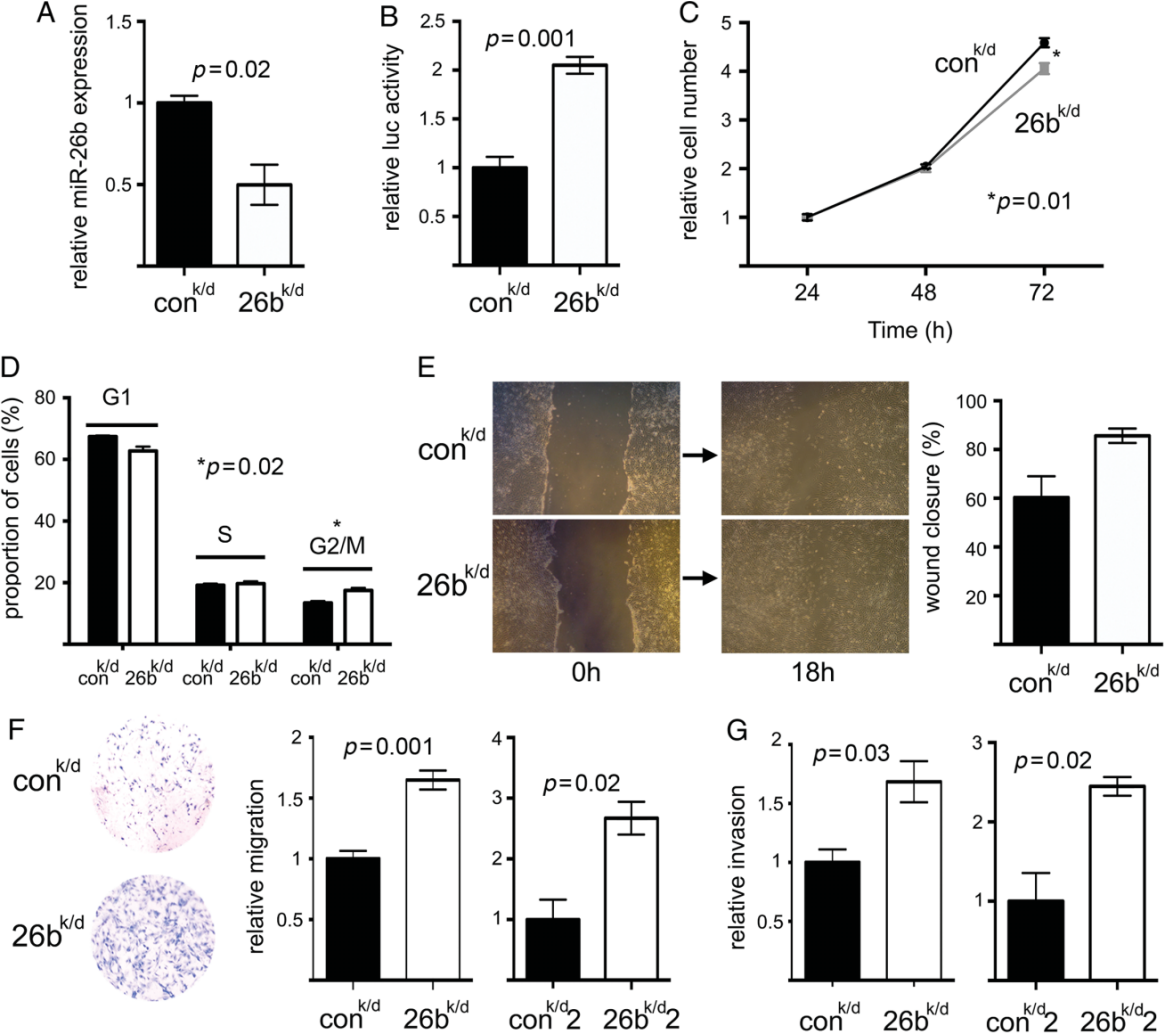
Stable down-regulation of miR-26b in breast fibroblasts inhibited growth but stimulated both migration and invasion. Immortalized breast fibroblasts were stably transduced to knock down miR-26b (26^k/d^) or with a control construct (con^k/d^). (A) MiR-26b expression was quantified in the two cell lines using qPCR (relative to RNU6B). Data are means of technical triplicates (± standard error) and are representative of duplicate experiments. (B) MiR-26b function was assessed as ratios of firefly to *Renilla* luciferase expression using a miR-26b target luciferase reporter (containing a perfect miR-26b binding site downstream of firefly luciferase and also coding for *Renilla* luciferase as an internal control). Cell lines were transfected with the reporter and dual luciferase assays were performed after 24 h. (C) Cell growth in the two cell lines was monitored using MTT assays over 72 h after initial seeding of equal numbers of cells. (D) Proportions of cells in G1, S, and G2/M phases of the cell cycle were determined in sub-confluent cultures using propidium iodide staining and flow cytometry. (E) Migration was determined in scratch-closure assays using digital imaging as the percentage scratch area remaining 18 h after scratch formation. Representative images are shown immediately after forming the scratch and at 18 h. (F) Migration was determined in trans-well assays by manual counting of cells that had passed through the membrane. A representative example of the trans-well migration result is shown. A second independent breast fibroblast line was also stably transduced (26^k/d^2 and con^k/d^2). Cells having migrated through the membranes were counted at 12 h for 26^k/d^ and con^k/d^ and at 24 h for 26^k/d^2 and con^k/d^2 (the second pair of transduced fibroblast lines migrated/invaded more slowly, reflecting variation between individual parental fibroblasts). (G) Invasion was assessed using trans-well assays by manual counting (at the same time points as F). Data in B–G are means of biological triplicates (± standard error) and are representative of duplicate (B, D–G) or triplicate (C) experiments.

To study the consequences of reduced miR-26b function, we stably knocked down miR-26b in breast fibroblasts using lentiviral vectors (which also conferred GFP expression). Fibroblasts were stably transduced to reduce miR-26b function (designated ‘26b^k/d^’), or were transduced with control virus (designated ‘control^k/d^’), and flow cytometry was used to enrich populations to maintain more than 90% transduced cells (GFP-positive). A two-fold reduction in miR-26b expression was observed by qPCR (Figure 5A; *p =* 0.02). As before, we were conscious that this might not accurately reflect functional knock-down; we therefore also performed luciferase reporter assays to assess miR-26b function. A single, fully complementary miR-26b binding site was cloned downstream of the luciferase reading frame in a reporter, and 26b^k/d^ or control^k/d^ cells were transfected with the reporter. Luciferase assays demonstrated that 26b^k/d^ cells allowed two-fold higher expression of a miR-26b target (Figure 5B; *p =* 0.001), indicative of reduced miR-26b function. We also examined the influences of miR-26b knock-down on cell growth/viability by both monitoring the growth of 26b^k/d^ and control^k/d^ cells (Figure 5C) and examining their cell cycle distributions (Figure 5D). In accordance with our previous findings (Figure 4A), we found that 26b^k/d^ cells grew slightly more slowly than control^k/d^ cells (*p =* 0.01 at 72 h). This appeared to relate to a cell cycle defect represented by an increase in cells in the G2/M phase (Figure 5D; *p =* 0.02). However, these growth and cell cycle defects are minor and may lack biological relevance. Next, we examined the migratory or invasive capacities of 26b^k/d^ and control^k/d^ cells using scratch-closure and trans-well migration assays, or trans-well Matrigel invasion assays. 26b^k/d^ cells showed increased migration in scratch-closure assays, although this fell just short of statistical significance (Figure 5E; *p =* 0.06). However, 26b^k/d^ cells demonstrated significantly enhanced migration and invasion in trans-well assays (Figure 5F, *p =* 0.001; Figure 5G, *p =* 0.03). A second independent immortalized breast fibroblast line was also transduced to knock down miR-26b (designated ‘26b^k/d^2’) or was control transduced (designated ‘control^k/d^2’); this also demonstrated significantly enhanced migration and invasion after miR-26b knock-down (Figure 5F, *p =* 0.02; Figure 5G, *p =* 0.02). We concluded that breast fibroblasts with reduced miR-26b, as seen in CAFs, exhibited reduced growth but increased motility and invasive capabilities.

### Reduced miR-26b activity in fibroblasts enhances migration/invasion of epithelial cancer cells

**Figure 6:**
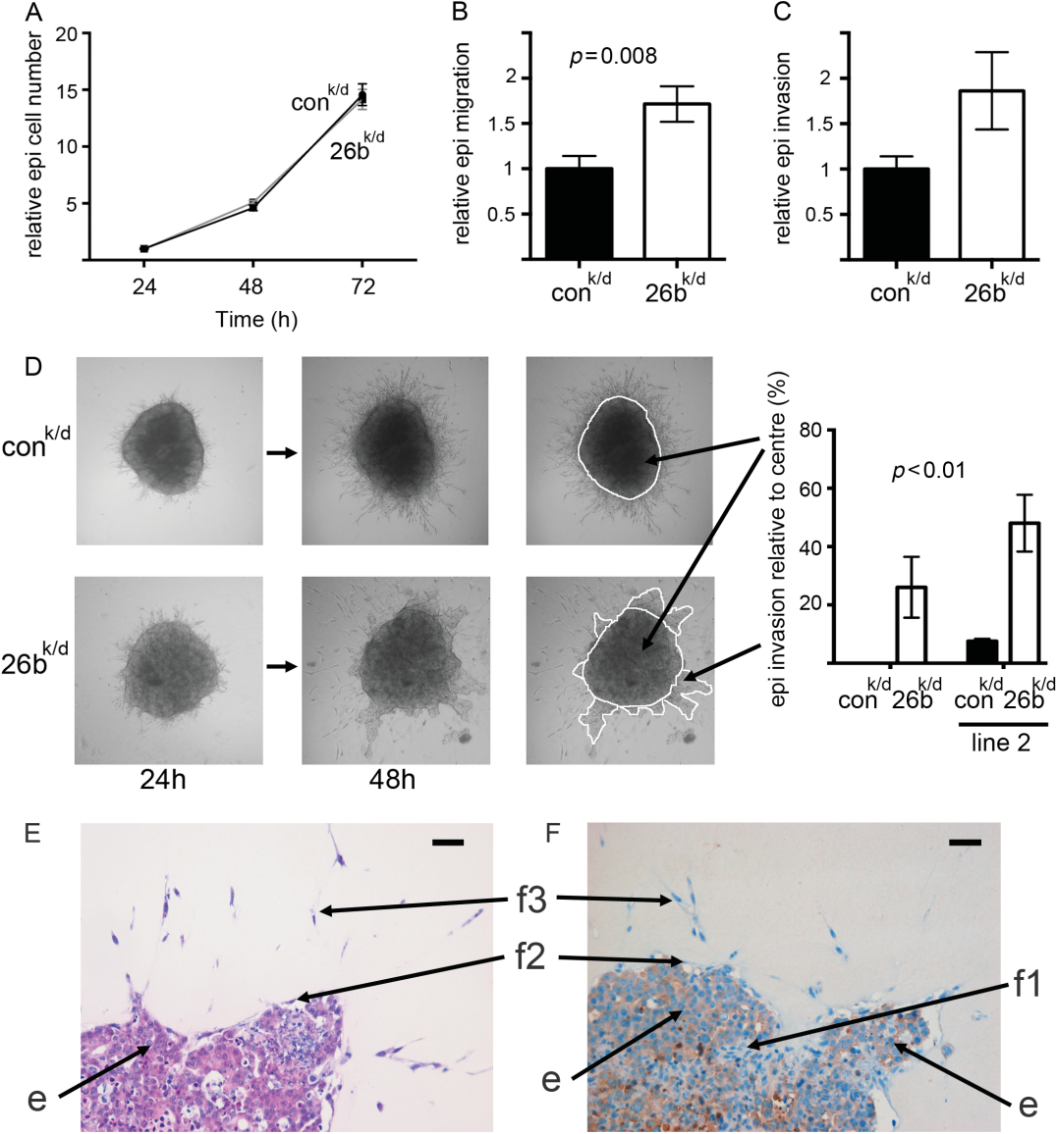
MCF7 cell migration and invasion are stimulated by breast fibroblasts with reduced miR-26b. MCF7 cells (luciferase-positive) were co-cultured with miR-26b knock-down (26^k/d^) or with control (con^k/d^) breast fibroblasts (seeding ratio of one epithelial cell to three fibroblasts). (A) MCF7 cell growth was monitored within co-cultures using luciferase assays over 72 h (entire co-cultures were lysed and luciferase activity, present within the epithelial cells only, was quantified). (B, C) Migration or invasion of MCF7 cells within co-cultures was assessed 24 h after seeding in trans-well assays using luciferase assays (cells having passed through the membrane were lysed and luciferase activity was quantified within the lysates). (D) MCF7 cells and fibroblasts were aggregated, forming three-dimensional spheroids, and were suspended in a collagen-I/Matrigel matrix for up to 48 h. Invasion of MCF7 cells away from the central spheroid was quantified as shown at 48 h. Assays were performed with 26^k/d^ or con^k/d^ fibroblasts and with equivalent lines derived from an independent breast fibroblast line (line 2). (E, F) Spheroids were formalin-fixed and paraffin-embedded. Sections were taken and stained using haematoxylin and eosin (E) or for epithelial cytokeratins (F). Scale bars (top right of each image) = 100 µm. Epithelial cells (cobblestone morphology/cytokeratin-positive) are labelled ‘e’, while populations of fibroblasts (elongated morphology/cytokeratin-negative) are labelled ‘f1’ (internal to spheroid outgrowth), ‘f2’ (surface of outgrowth), and ‘f3’ (within matrix radiating from spheroid). Data are means of at least biological triplicates (± standard error) and are representative of duplicate (B–D) or triplicate (A) experiments.

We were especially interested to determine whether miR-26b in breast fibroblasts could modify epithelial cancer cell behaviour. We co-cultured either 26b^k/d^ or control^k/d^ fibroblasts with MCF7 epithelial cancer cells. We used MCF7 cells that stably express luciferase in order to quantify them separately within co-cultures. As expected, the presence of fibroblasts enhanced MCF7 growth (see Figure 2), but 26b^k/d^ and control^k/d^ fibroblasts did not have differential influences (Figure 6A). This experiment was performed seeding epithelial cells and fibroblasts at a ratio of 1 : 3 in accordance with published literature 6; differential influences of 26b^k/d^ and control^k/d^ fibroblasts on epithelial growth were also not seen at other ratios (Supplementary Figure 2). Next, migration and invasion assays were performed with MCF7/fibroblast co-cultures (seeding ratios of 1 : 3). In trans-well assays, MCF7 cells demonstrated significantly enhanced migration in the presence of 26b^k/d^ fibroblasts (Figure 6B; *p =* 0.008), although a trend for enhanced invasion fell short of statistical significance (Figure 6C). This enhanced migration of MCF7 cells was not reproduced when the fibroblasts were seeded separately in the lower chambers of the trans-wells (Supplementary Figure 3). Invasion assays were also performed using three-dimensional spheroids, which better reflect *in vivo* interactions between cells and the microenvironment 34. MCF7 cells were aggregated with 26b^k/d^ or control^k/d^ fibroblasts, or with each of the second pair of transduced fibroblasts, 26b^k/d^2 or control^k/d^2, before being encased in collagen-I/Matrigel matrix and incubated for up to 48 h. Invasion into the matrix of large groups of cells was dramatically enhanced by the 26b^k/d^ fibroblasts (Figure 6D; *p =* 0.01) compared with controls; indeed, these outgrowths were absent in the presence of one control line. These striking outgrowths were characterized in more detail by staining with haematoxylin and eosin (Figure 6E) or for epithelial cytokeratins (Figure 6F). Outgrowths contained predominantly epithelial cells (based on morphology and positive cytokeratin expression; labelled ‘e’) with some internal fibroblasts (based on morphology and/or negative cytokeratin expression; labelled ‘f1’). Fibroblasts were mainly visible on the external surface of outgrowths (labelled ‘f2’) and radiating through the matrix from the spheroid (labelled ‘f3’).

### MiR-26b regulates multiple molecular pathways in breast fibroblasts

**Figure 7:**
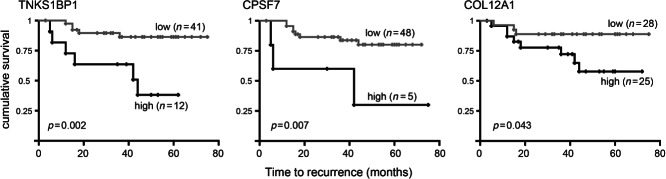
Stromal expression of inferred miR-26b targets predicts breast cancer recurrence. Correlations between high and low (as defined using ROC analyses) stromal expression of *TNKS1BP1*, *CPSF7* or *COL12A1* and breast cancer recurrence were tested using Kaplan–Meier analyses, using publicly available mRNA expression array data from laser capture micro-dissected stromal breast cancer tissue from 53 breast cancers 5, mined using the Oncomine platform (https://www.oncomine.org).

Our next aim was to determine the molecular pathways responsible for the altered behaviour of 26b^k/d^ fibroblasts. Initially, we identified potential direct miR-26b targets using bioinformatics (TargetScan; http://www.targetscan.org); these predictions suggested that miR-26b might target more than 100 different transcripts. In order to identify pathways altered in breast fibroblasts, we carried out proteomic comparisons of 26b^k/d^ and control^k/d^ fibroblasts. Label-free protein mass spectrometry was performed on triplicate flasks of both cell types (dataset—Supplementary Data 3). As expected, expressions in the triplicates were highly related, with mean Spearman's rho coefficients for pairwise comparisons between triplicates of 0.83 and 0.85 for 26b^k/d^ and control^k/d^ cells, respectively (*p* < 0.001). We identified proteins that were significantly differentially expressed between the lines. Three hundred and sixty proteins (of a total of 3369 detected) were differentially expressed (Student's *t*-test; *p* < 0.05). When multiple testing was taken into account using a false discovery rate (FDR) threshold of 0.1, this was reduced to 11 proteins (Table2). In order to determine molecular pathways that were deregulated in 26b^k/d^ cells, we subjected the protein lists to gene ontology analyses (ToppGene Suite; http://toppgene.cchmc.org/). The list of 360 proteins was significantly enriched for genes involved in glycolysis and TCA cycle (nine genes from 35 in the genome; *p* < 0.01) and in cytoskeletal regulation by Rho GTPases (14 from 72; *p* < 0.0001) (Supplementary Table 2). For identification of potential direct miR-26b targets, we limited our analyses to changes in expression identified at an FDR of less than 0.1. We examined whether the 11 differentially expressed proteins were predicted miR-26b targets and whether this correlated with up- or down-regulation; up-regulation in 26b^k/d^ cells was expected for direct miR-26b targets. Five proteins were up-regulated in 26b^k/d^ cells, three of which were predicted targets, while six were down-regulated, none of which were predicted targets (Table2). This distribution of predicted targets supports the conclusion that these three predictions are true direct targets. These are tankyrase 1 binding protein 1 (*TNKS1BP1*), cleavage and polyadenylation-specific factor 7 (*CPSF7*), and collagen type XII alpha 1 (*COL12A1*). The functional relevance of these three molecules was examined further by mining publicly available data. Expression data are available for 53 breast cancers from which cancer stroma was isolated by LMD and stromal mRNA expression levels were profiled using expression arrays 5. We tested whether stromal expression of *TNKS1BP1*, *CPSF7* or *COL12A1* was associated with differences in time to recurrence. Expression was dichotomized using Receiver Operator Curve (ROC) analysis in order to allow Kaplan–Meier analyses of groups with high and low expression. For each gene, high expression, as seen in 26b^k/d^ cells, was significantly associated with increased rates of recurrence (Figure 7; log rank, *TNKS1BP1 p =* 0.002, *CPSF7 p =* 0.007, *COL12A1 p =* 0.043), implicating them, and by inference miR-26b, as stromal determinants of breast cancer outcome.

**Table 2:** Proteins differentially expressed between control^k/d^ and 26^k/d^ fibroblasts (false discovery rate < 0.1). Immortalized breast fibroblasts were virally transduced to reduce expression of miR-26b (26^k/d^) or were control transduced (control^k/d^). Label-free quantitative protein mass spectrometry was performed on triplicate flasks of the two cell lines. Fold changes were calculated using mean expression levels in triplicate control^k/d^ samples and triplicate 26^k/d^ samples

Gene	Regulation/protein fold change*	Predicted target
*TMEM119*	Down	No
*NUDCD3*	Down	No
*TPM2*	Down	No
*FAM3C*	Down	No
*CLTB*	Down	No
*THOC5*	Down	No
*USP19*	Up	No
*APIP*	Up	No
*TNKS1BP1*	Up/3.32	Yes
*CPSF7*	Up/4.35	Yes
*COL12A1*	Up/7.20	Yes

* Fold changes are shown only when the protein was detected in both cell lines–when it was not detected in either line, only the direction of deregulation is indicated.

## Discussion

CAFs are known to differ from NFs in their expression profiles and, consequently, in their behaviour and influences on epithelial cancer cells. However, the molecular basis of these expression differences is poorly understood, with signalling from cancer and other stromal cells 35, as well as epigenetic deregulation 10, implicated as key influences. We examined whether miRNA expression differs substantially between NFs and CAFs and whether these differences regulate fibroblast behaviour. We focused on miR-26b, since it was deregulated (down-regulated in CAFs) to the greatest extent in our screens (Table1). Down-regulation of miR-26b has been observed previously in various cancers, including glioma 36, head/neck/oral cancer 37, hepatocellular cancer 38, and breast cancer 39, and has generally correlated with higher grade or more aggressive cancer types. This conclusion is supported by the fact that low miR-26b expression correlated significantly with poor cancer survival in hepatocellular carcinoma 38, and in breast cancer in our analysis of publicly available miRNA expression data (Supplementary Figure 4). It should be emphasized that all of these studies analysed expression in cell populations that included both cancer and stromal cells; therefore it is not possible to be certain of the cell type in which miR-26b is expressed and functional. An exception to this is a recent analysis of miRNA expression in six paired primary cultures of NFs and CAFs from breast cancers, in which miR-26b was identified as down-regulated in CAFs 20, in accordance with our findings, although it is important to note that this study included no assessments of the function or targets of miR-26b.

At least 14 different direct targets for miR-26b have been suggested in the literature (see Supplementary Table 3) in a range of other cell types and supported by differing levels of proof. The vast majority of these potential target proteins were not detected in our proteomic analyses, but those that were showed no evidence of significant differential expression between 26b^k/d^ and control^k/d^ cells. This is compatible with the suggestion that miR-26b targets multiple transcripts and that these vary with cell type. We identified three miR-26b targets in breast fibroblasts and established that their expression in this compartment was significantly associated with survival from breast cancer (Figure 7). These molecules are functionally diverse: TNKS1BP1 is involved in telomere maintenance 40; CPSF7 regulates polyadenylation 41; and COL12A1 is an extracellular matrix component 42. This diversity hints at the potential for miR-26b to have broad-ranging influences on cellular physiology, as has been seen with some other miRNAs 43. Importantly in this context, we analysed downstream effects of manipulating miR-26b at a wider proteome level. We identified two significantly deregulated pathways: glycolysis/TCA cycle; and cytoskeletal regulation by Rho GTPases. For glycolysis/TCA, all genes were up-regulated in 26b^k/d^ fibroblasts and they included enzymes that catalyse five of the nine main steps of glycolysis. Similar up-regulation of glycolytic enzymes has recently been reported in bladder cancer stroma 44. These observations are potentially compatible with the ‘reverse Warburg effect’, as described by Lisanti and co-workers 45, in which CAFs carry out aerobic glycolysis, thereby producing lactate and pyruvate that are used, in part, by neighbouring cancer cells. However, published work suggests that this results in enhanced cancer cell growth 46, which we did not find (Figure 6A), rather than the enhanced cancer cell migration/invasion that we saw (Figures 6B–6F). With respect to the second pathway, the influences of Rho GTPases on cytoskeletal dynamics and cellular motility are well established 47. Notably, Rho signalling has been implicated in CAF-mediated remodelling of the tumour microenvironment, leading to enhanced invasion of cancer cells 48,49, a model discussed further below.

We have shown that reduced miR-26b can enhance breast fibroblast migration and invasion (Figures 4 and 5) and that this, in turn, can stimulate migration and invasion of epithelial cancer cells in the context of epithelial/fibroblast co-cultures (Figure 6). At least two models have been proposed to explain this stromal–epithelial cross-talk. Firstly, fibroblasts secrete soluble paracrine factors that stimulate epithelial migration/invasion directly. Secondly, fibroblasts modify the structural microenvironment, making it more permissive for epithelial migration/invasion. From our data, we believe that a soluble paracrine factor that acts directly on the epithelial cells is less likely, since we were unable to recapitulate the influence of 26b^k/d^ fibroblasts on epithelial cells when the cells were physically separated in culture (Supplementary Figure 3). Therefore, our preferred model is one that involves modification of the structural microenvironment. Potentially, enhanced epithelial invasion could be a passive phenomenon where epithelial invasion occurs simply by following the passage of fibroblasts through the matrix, making use of the ‘holes’ that remain behind the fibroblasts; in this case, fibroblasts would be simply removing the barrier of the matrix that inhibits epithelial movement. However, our data indicate that the fibroblasts' influence is more complex. 26b^k/d^ fibroblasts significantly stimulated the migration of epithelial cells in trans-wells lacking matrix, where there was no barrier for the fibroblasts to remove and no matrix in which fibroblasts could leave these ‘holes’ (Figure 6B). In addition, control^k/d^ fibroblasts invaded from spheroids, albeit to a lesser degree than 26b^k/d^ fibroblasts, presumably leaving the ‘holes’ in the matrix, but epithelial invasion was hardly detectable at all. We interpret this to suggest that active stimulation of epithelial migration/invasion through stimulatory modifications to the matrix is a component of the 26b^k/d^ fibroblasts' influence. Fibroblasts enhance the invasion of squamous cell carcinoma cells in a similar way, with both matrix ‘holes’ and fibroblast deposition of the matrix components fibronectin and tenascin-C implicated as potential stimulatory mediators of epithelial invasion 49. In this case, RhoA signalling in the fibroblasts was found to be important for both hole formation and matrix deposition 49, which correlates with our observation of deregulation of the Rho pathway. The roles of fibronectin and tenascin-C in stimulating this migration/invasion remain to be fully elucidated. COL12A1 presents a further candidate molecule for roles in making the matrix permissive for migration/invasion as it is known to organize and stabilize matrix fibrils of collagen type I 50, a main matrix component in our spheroid assay. In addition, increased COL12A1 expression has recently been noted at colorectal cancer invasive fronts, implicating COL12A1 in invasion 42.

## Supporting Information On The Internet

The following supporting information may be found in the online version of this article.

Supplementary materials and methods.

Table S1 Pathological features of the breast cancer cases included in this study.

Table S2 Components of pathways identified by Gene Ontology analyses as enriched in the proteins differentially expressed between control^k/d^ and 26^k/d^ fibroblasts.

Table S3 Published miR-26b targets.

Figure S1 Transient miR-26b up-regulation in breast fibroblasts induced dramatic growth inhibition and apoptosis.

Figure S2 MiR-26b knock-down or control breast fibroblasts do not have differential influences on MCF7 cell growth at a range of different seeding densities.

Figure S3 MCF7 cell migration is not stimulated by breast fibroblasts with reduced miR-26b when the cells are seeded in separate chambers of trans-wells.

Figure S4 Expression of miR-26b predicts breast cancer recurrence.

Supplementary Data 1 Microarray data of miRNA expression levels from NFs and CAFs and normal and cancer epithelial cells from FFPE breast tissue.

Supplementary Data 2 MiRNAs consistently up- or down-regulated in CAFs compared with NFs in both FFPE breast tissue and the tissue culture model.

Supplementary Data 3 Protein expression levels in triplicate samples of 26^k/d^ (T1, T2, and T3) and control^k/d^ (C1, C2, and C3) fibroblasts as determined by mass spectrometry.
