# Supplementary material for: MiR-26b is down-regulated in carcinoma-associated fibroblasts from ER-positive breast cancers leading to enhanced cell migration and invasion
Source: J Pathol. 2013 Oct 9;231(3):388–99. doi: 10.1002/path.4248 (PMC4030585; doi:10.1002/path.4248)
Supplement: Supplementary file 1 [file path0231-0388-sd1.doc]

**SUPPORTING INFORMATION**

**Supplementary materials and methods**

***Cell culture and flow-cytometry***

Cell line identities were confirmed by annual STR profiling (Powerplex-16 reagents; Promega; Fitchburg, WI, USA). Tissue culture reagents were supplied by Invitrogen (Paisley, UK), except for foetal calf serum (“Hyclone”, Fisher Scientific; Waltham, MA, USA). All cell lines were maintained in D-MEM, 10% serum in humidified air/5% CO_2_ at 37^0^C. Bi-monthly mycoplasma checks (MycoAlert, Mycoplasma detection assay; Lonza; Basel, Switzerland) were consistently negative for immortal lines and were not performed for short-term primary cultures. Fluorescence-activated cell sorting of GFP-positive cells used untranfected/non-transduced cells as negative controls to define gating. Cells to be sorted were suspended in medium/2% serum and filtered through a through 70µm cell strainer, and were sorted into medium/20% serum.

***Laser micro-dissection (LMD)***

Formalin-fixed paraffin-embedded (FFPE) tissue blocks containing breast cancer tissues or matched normal breast tissues (>1cm from tumours) were available from 15 patients treated for primary breast cancer at Leeds Teaching Hospitals NHS Trust (see Table S1 for characteristics of the tumours). For each archival block up to four 10µm sections were prepared on uncoated glass slides (Menzel-Glaser; Braunschweig, Germany). These were placed on a hot plate (55^0^C) for 5-8min to fix tissue to the slides (duration was increased from 5min if considerable fat content was evident visually). Slides were allowed to cool to room temperature and were transferred into xylene (at 60^o^C; 3min), and then 100% ethanol (room temperature; 3min). Slides were air dried (1min), stained with 1% toludine blue (30s), and washed with 100% ethanol. Tissues were visualised using the Zeiss/PALM Laser Dissection Microscope (Oberkochen, Germany) using 100x or 200x magnification. Epithelial and stromal compartments were identified from their morphology and digitally marked by ETV (an experienced and specialist breast histopathologist). Areas selected were devoid of visible cells other than target cells and were not necrotic, as determined by thorough visual examination of both the section to be micro-dissected (stained with toluidine blue) and an adjacent section (stained with hematoxylin and eosin). For each compartment 5-10mm^2^ was micro-dissected. Areas of vascularity, inflammation and necrosis were avoided. Energy settings for the dissection laser varied between individual samples: settings allowing successful dissection were obtained separately for every tissue by trial and error.

***Microarray analyses and quantitative PCR (qPCR)***

Microarray analyses of miRNA expression were performed using human miRNA v2 arrays and system v1.7 reagents (Agilent; Santa Clara, CA, USA). 100ng total RNA was treated with 0.7μl calf intestinal phosphatase (Clontech; Mountain View, CA, USA) followed by 5μl of 100% DMSO (Sigma; Poole, UK). 3μl Cyanine dye (Cy3) was ligated to RNAs using 2μl T4 RNA Ligase (New England Biolabs; Ipswich, MA, USA) for 2 hours at 16^0^C. The labelled RNAs were purified using Micro Bio-Spin Columns with Bio-Gel (Bio-Rad; Hercules, CA, USA). Hybridisation with human miRNA v2 arrays (Design ID 019118) was carried out at 55^o^C for 20h in blocking agent (Agilent; Santa Clara, CA, USA). Arrays were washed and scanned according to the manufacturer’s protocols. Features extraction software (v9.5.3; Agilent; Santa Clara, CA, USA) was used to probe the data. Baseline thresholding of Signal values were log2 transformed and 75^th^ percentile normalization performed. The raw and normalised data are presented as Data S1. qPCR analyses were performed on 7500 or 7900HT machines. A Megaplex pre-amplification step as used when working from FFPE material (Life Technologies; Carlsbad, CA, USA). Relative miRNA expression was determined using normalisers RNU6B and RNU48. Relative mRNA expression was determined normalised to GAPDH.

***Reverse transfection of fibroblasts***

Reverse transfection of pre-/anti-miR (Ambion; Carlsbad, CA, USA) and/or plasmids was performed using HiPerFect (Qiagen; Hilden, Germany). For each well of a 24-well plate, transfection complexes were prepared by incubating plasmid DNA (up to 1.5 µg) or RNA (final concentrations of up to 50nM pre- or anti-miRs) with 3µl of HiPerFect made up to 50µl with serum free medium (15min, room temperature). 15000 fibroblasts suspended in serum-free medium were added to each well and incubated (37^0^C, 5min). Transfection complexes were added to wells and cells cultured at 37^0^C. Medium was changed to serum containing medium after 18h.

***26b^k/d^ and control^k/d^ fibroblasts***

Stable miR-26b/control ‘knock-down’ was performed using pmiRZIP lentiviral vectors (System Biosciences; Mountain View, CA, USA). pmiRZIP-26b and pmiRZIP-control plasmids were purchased. 2μg miRZIP plasmid DNA was transfected with pPACK-H1 Lentivector packaging plasmids into 293TN packaging cells (System Biosciences; Mountain View, CA, USA) using Lipofectamine Plus reagent (Invitrogen; Paisley, UK). Supernatants containing pseudoviral particles were taken after 48h and any 293TN cells were removed by centrifugation. 1x10^5^ fibroblasts were seeded in each well of 12-well plates and were incubated overnight (37^0^C). Medium was then replaced with 1ml of fresh medium containing 5μg/ml polybrene (Sigma; Poole, UK). 200μl of pseudoviral particle-containing supernatant was added to each well and incubated overnight (37^0^C). Medium was replaced and cells were split as determined by growth of individual cultures. Initial transduction efficiencies ranged from 80% to 90% as determined by % GFP-positivity using flow-cytometry (pmiRZIP vectors also encode stable over-expression of GFP). Fluorescence-activated cell sorting was used to establish and maintain populations as >90% GFP-positive.

***Assays of cellular behaviour***

MTT assays were used to quantify viable cells, propidium iodide/flow-cytomtery was used quantify cells in each cycle phase, and FITC-Annexin V/7-AAD staining were used to detect cells in early (Annexin V positive, 7-AAD negative) or late (Annexin V positive, 7-AAD positive) apoptosis. Migration and invasion were assessed using trans-well assays (8μm pore size; BD Bioscience; Franklin Lakes, NJ, USA), with either uncoated (migration) or Matrigel-coated (invasion) inserts [16]. Test cell line(s) were seeded in upper chambers without serum while lower chambers contained either fibroblasts in medium/10% serum or medium/10% serum alone. Cells migrating/invading were quantified by counting cells on lower membrane surfaces after staining with toluidine blue (1 central and 4 peripheral fields at 200x magnification) or, in the case of luciferase-positive MCF7 cells, using luciferase assays (Promega; Fitchburg, WI, USA). In the case of quantification using luciferase assays, cells that had not passed through the membrane were thoroughly removed from upper surface using cotton swabs. Each membrane was then incubated in 300μl of passive lysis buffer (20 min, room temperature) and luminescence assayed using the LARII component of the Dual Luciferase Reporter System (Promega; Fitchburg, WI, USA) following the manufacturer’s protocol. For scratch-closure assays, cells were grown to 90% confluence and monolayers were scratched using P200 pipette tips and reference marks were made in the field to allow longitudinal imaging of the same field for up to 48h. Cells were imaged using an E1000 Eclipse microscope (Nikon; Tokyo, Japan) and closure was analysed using Photoshop (Abode; San Jose, CA, USA). Spheroid co-culture invasion assays were carried out using modifications of a previously described method [27]. Equal numbers (12500) of fibroblasts and epithelial cells were suspended in a 25μl drop of medium and were cultured in the centre of a Mat-Tek dish and inverted overnight. 300μl 4mg/ml collagen-1, 2.2mg/ml Matrigel was added to each well of a 24-well plate (37^0^C; 1h). Cell aggregates from overnight cultures were placed on top of these gels and 200μl of collagen-1/Matrigel mix was added above (37^0^C; 1h). 300μl culture medium was added to each well. Spheroids were imaged using an E1000 Eclipse microscope (Nikon; Tokyo, Japan). Invasion was quantified using a modification of the method previously described [51]. The central spheroidal area and the radial extent (peripheral circumferential area) of the spheroid were calculated using Photoshop (Abode; San Jose, CA, USA) and invasion was quantified as circumferential area relative to central spheroidal area. For analysis of spheroid histology, matrix gels containing spheroids were removed from wells and immersed in 4% formalin (16h, room temperature) before being paraffin-embedded. 5µm sections were stained with haematoxylin/eosin or treated for immunohistochemistry using an IntelliPATH automated stainer (Menarini; Florence, Italy). Sections were dewaxed and antigens were retrieved in Access Revelation Solution in the Access retrieval unit (Menarini; Florence, Italy). Slides were stained using the IntelliPATH running the standard X-Cell plus protocol with rabbit anti-cytokeratin (Ab9377; Abcam; Cambridge, UK) diluted at 1:100 in Zymed antibody diluent (Invitrogen; Paisley, UK). Slides were counterstained in haematoxylin, mounted in DPX (Sigma; Poole, UK) and images taken using the E1000 Eclipse microscope (Nikon; Tokyo, Japan). The pmiRGLO vector and Dual Luciferase Reporter System reagents were purchased from Promega (Fitchburg, WI, USA). Oligonucleotides encoding a perfect miR-26b target site (top strand: 5’-AACCTATCCTGAATTACTTGAAG-3’; bottom strand: 5’-TCGACTTCAAGTAATTCAGGATAGGTTAGCT-3’) were cloned into the vector *Sac*I/*Sal*I. Dual luciferase assays (firefly luciferase under the influence of the test miR and *renilla* luciferase as an internal control) were performed from triplicate transfections using the manufacturer’s protocols.

***Protein mass spectrometry***

Lysates were prepared from cell pellets and processed by filter-aided sample preparation as described previously using trypsin [29]. Peptides were separated by capillary liquid chromatography (LC) and analyzed by tandem mass spectrometry (MS/MS) using RSLCnano system (Dionex/Thermo Scientific; Waltham, MA, USA) and LTQ-Orbitrap Velos mass spectrometer (Thermo Scientific; Waltham, MA, USA). The samples were injected onto a 20cm capillary column (inner diameter 75µm, 3.5µm Kromasil C_18_ media). Total acquisition time was 240min, the main gradient being 4–25% ACN in 0.1% formic acid at 400nl/min. MS scans were acquired in the orbitrap with resolution of 60000. Up to 20 ions per scan were fragmented in the ion-trap. The data search against IPI Human 3.87 database and label-free quantitation (LFQ) were performed using MaxQuant 1.2.2.5 [30].

**References**

**(Note: reference numbers correspond to reference list in main article)**

16. Ma L, Teruya-Feldstein J, Weinberg RA. Tumour invasion and metastasis initiated by microRNA-10b in breast cancer. *Nature* 2007; **449**: 682-688.

27. Nowicki MO, Dmitrieva N, Stein AM*, et al.* Lithium inhibits invasion of glioma cells; possible involvement of glycogen synthase kinase-3. *Neuro Oncol* 2008; **10**: 690-699.

29. Wisniewski JR, Zougman A, Nagaraj N*, et al.* Universal sample preparation method for proteome analysis. *Nature Methods* 2009; **6**: 359-362.

30. Cox J, Matic I, Hilger M*, et al.* A practical guide to the MaxQuant computational platform for SILAC-based quantitative proteomics. *Nature Protocols* 2009; **4**: 698-705.

51. Hattermann K, Held-Feindt J, Mentlein R. Spheroid confrontation assay: a simple method to monitor the three-dimensional migration of different cell types *in vitro*. *Ann Anat* 2011; **193**: 181-184.

52. Lyng MB, Laenkholm AV, Sokilde R*, et al.* Global microRNA expression profiling of high-risk ER+ breast cancers from patients receiving adjuvant tamoxifen mono-therapy: a DBCG study. *PloS One* 2012; **7**: e36170.

| **Usage** | **Type** | **Grade** | **LN status (+/-)** | **ER status (+/-)** | **Her2 status (+/-)** |
| --- | --- | --- | --- | --- | --- |
| Microarrays | ductal-NST | 2 | - | + | - |
|  |  |  |  |  |  |
| qPCR 1 | ductal-NST | 2 | - | + | - |
| qPCR 2 | ductal-NST | 2 | - | + | - |
| qPCR 3 | ductal-NST | 2 | - | + | - |
| qPCR 4 | ductal-NST | 2 | - | + | - |
| qPCR 5 | ductal-NST | 2 | - | + | - |
| qPCR 6 | ductal-NST | 2 | - | + | - |
| qPCR 7 | ductal-NST | 2 | - | + | - |
| qPCR 8 | ductal-NST | 2 | - | + | - |
| qPCR 9 | ductal-NST | 2 | - | + | - |
| qPCR 10 | ductal-NST | 2 | - | + | - |
| qPCR 11 | ductal-NST | 2 | - | + | - |
| qPCR 12 | ductal-NST | 2 | - | + | - |
| qPCR 13 | ductal-NST | 2 | - | + | - |
| qPCR 14 | ductal-NST | 2 | - | + | - |
|  |  |  |  |  |  |
| 1^o^ cultures 1 | ductal-NST | 2 | - | + | - |
| 1^o^ cultures 2 | ductal-NST | 3 | - | + | - |
| 1^o^ cultures 3 | ductal-NST | 1 | - | + | - |
| 1^o^ cultures 4 | Mixed | 1 | unknown | + | + |

**Table S1.** Pathological features of the breast cancer cases included in this study. NST: no special type; LN: lymph node; ER: estrogen receptor alpha.

|  | **Gene** | **Gene name** | **Fold Change** |
| --- | --- | --- | --- |
| **GROUP 1: cytoskeletal regulation by Rho GTPases** | | |  |
| 1 | ACTB | actin, beta | 2.58 |
| 2 | ACTG1 | actin, gamma 1 | 2.58 |
| 3 | ACTBL2 | actin, beta-like 2 | 4.39 |
| 4 | ARHGAP1 | Rho GTPase activating protein 1 | 2.53 |
| 5 | PAK1 | p21 protein (Cdc42/Rac)-activated kinase 1 | 1.52 |
| 6 | MYH10 | myosin, heavy chain 10, non-muscle | 5.45 |
| 7 | ARPC1A | actin related protein 2/3 complex, subunit 1A, 41kDa | down* |
| 8 | CFL2 | cofilin 2 (muscle) | 3.59 |
| 9 | CFL1 | cofilin 1 (non-muscle) | 1.69 |
| 10 | TUBB6 | tubulin, beta 6 class V | 2.91 |
| 11 | PFN1 | profilin 1 | 1.72 |
| 12 | ARPC5 | actin related protein 2/3 complex, subunit 5, 16kDa | down* |
| 13 | ROCK2 | Rho-associated, coiled-coil containing protein kinase 2 | down* |
| 14 | STMN1 | stathmin 1 | 1.69 |
|  |  |  |  |
| **GROUP 2: glycolysis/gluconeogenesis and TCA** | | |  |
| 1 | ALDOA | aldolase A, fructose-bisphosphate | 1.46 |
| 2 | SDHA | succinate dehydrogenase complex, subunit A, flavoprotein (Fp) | 2.68 |
| 3 | MDH1 | malate dehydrogenase 1, NAD (soluble) | 2.15 |
| 4 | PGK1 | phosphoglycerate kinase 1 | 2.02 |
| 5 | PGAM1 | phosphoglycerate mutase 1 (brain) | 2.06 |
| 6 | IDH1 | isocitrate dehydrogenase 1 (NADP+), soluble | 2.45 |
| 7 | DLST | dihydrolipoamide S-succinyltransferase (E2 component of 2-oxo-glutarate complex) | 1.92 |
| 8 | ENO1 | enolase 1, (alpha) | 1.84 |
| 9 | GAPDH | glyceraldehyde-3-phosphate dehydrogenase | 1.62 |

**Table S2.** Components of pathways identified by Gene Ontology analyses as enriched in the proteins differentially expressed between control^k/d^ and 26^k/d^ fibroblasts. Gene names and fold differences in expression are shown. Fold changes were calculated using mean expression levels in triplicate control^k/d^  samples and triplicate 26^k/d^ samples. *Fold changes are shown only when the protein was detected in both cell lines – when it was not detected in either line only the direction of deregulation is indicated.

| **Target** | **Reference** |
| --- | --- |
| GATA4 | Han M, et al. (2012) GATA4 expression is primarily regulated via a miR-26b-dependent post-transcriptional mechanism during cardiac hypertrophy. Cardiovasc Res 93, 645-654 |
| PTEN | Palumbo T, et al. (2013) Functional screen analysis reveals miR-26b and miR-128 as central regulators of pituitary somatomammotrophic tumor growth through activation of the PTEN-AKT pathway. Oncogene 32, 1651-9 |
| ATF2 | Arora H, et al. (2011) Coordinated regulation of ATF2 by miR-26b in gamma-irradiated lung cancer cells. PLoS One 6, e23802 |
| ATM | Lin F, et al. (2012) miR-26b promotes granulosa cell apoptosis by targeting ATM during follicular atresia in porcine ovary. PLoS One 7, e38640 |
| CTDSP2 | Dill H, et al. (2012) Intronic miR-26b controls neuronal differentiation by repressing its host transcript, ctdsp2. Genes Dev 26, 25-30 |
| BDNF | Caputo V, et al. (2011) Brain derived neurotrophic factor (BDNF) expression is regulated by microRNAs miR-26a and miR-26b allele-specific binding. PLoS One 6, e28656 |
| COX2 | Ji Y, et al. (2010) MiRNA-26b regulates the expression of cyclooxygenase-2 in desferrioxamine-treated CNE cells. FEBS Lett 584, 961-967 |
| DNMT3b | Sandhu R, et al. (2012) Loss of post-transcriptional regulation of DNMT3b by microRNAs: a possible molecular mechanism for the hypermethylation defect observed in a subset of breast cancer cell lines. Int J Oncol 41, 721-732 |
| LEF1 | Zhang Z, et al. (2010) MicroRNAs regulate pituitary development, and microRNA 26b specifically targets lymphoid enhancer factor 1 (Lef-1), which modulates pituitary transcription factor 1 (Pit-1) expression. J Biol Chem. 285, 34718-34728 |
| EphA2 | Wu N, et al. Role of microRNA-26b in glioma development and its mediated regulation on EphA2. PLoS One 6, e16264 |
| SLC7A11 | Liu XX, et al. (2011) MicroRNA-26b is underexpressed in human breast cancer and induces cell apoptosis by targeting SLC7A11. FEBS Lett 585(9):1363-1367 |
| EZH2 | Koh CM, et al. (2011) Myc enforces overexpression of EZH2 in early prostatic neoplasia via transcriptional and post-transcriptional mechanisms. Oncotarget 2, 669-683 |
| cdc6  cyclin E1 | Zhu Y, et al. (2012) MicroRNA-26a/b and their host genes cooperate to inhibit the G1/S transition by activating the pRb protein. Nuc Acids Res 40, 4615-4625 |

**Table S3.** Published miR-26b targets.

**Supplementary Figure and Data Legends**

**Figure S1.** Transient miR-26b up-regulation in breast fibroblasts induced dramatic growth inhibition and apoptosis. Immortalised breast fibroblasts were transiently transfected with pre-miR-26b molecules or with control anti-miRs. A) Cell growth/viability was monitored using MTT assays over 72h. B) Apoptosis in transfected cultures was quantified using Annexin V/7-AAD staining and flow cytometry (data shown combine both early and late apoptosis). C) MiR-26b expression was quantified 48h after transfection using qPCR (relative to RNU6B). Data in A and B are means of biological triplicates (±standard error) and are representative of duplicate experiments. Data in C are means of technical triplicates (±standard error) and are representative of duplicate experiments (note the technical error is too small to be visible at this scale).

**Figure S2.** MiR-26b knock-down or control breast fibroblasts do not have differential influences on MCF7 cell growth at a range of different seeding densities. MCF7 cells (luciferase positive) were co-cultured with miR-26b knock-down (26^k/d^) or with control (con^k/d^) breast fibroblasts at 3 different seeding ratios, keeping the number of epithelial cells constant. MCF7 cell numbers were monitored within the co-culture using luciferase assays after 72h. Differences between 26^k/d^ and con^k/d^ are not significant in each case. Data are means of biological triplicates (±standard error) and are representative of duplicate experiments.

**Figure S3.** MCF7 cell migration is not stimulated by breast fibroblasts with reduced miR-26b when the cells are seeded in separate chambers of trans-wells. MCF7 cells (luciferase positive) were seeded in the upper chamber of trans-wells. MiR-26b knock-down (26^k/d^) or control (con^k/d^) breast fibroblasts were seeded in the lower chamber of trans-wells. Migration of MCF7 cells through the trans-well membrane was assessed 48h after seeding using luciferase assays (cells having passed through the membrane were lysed and luciferase activity quantified within the lysates). Data are means of biological triplicates (±standard error) and are representative of duplicate experiments.

**Figure S4.** Expression of miR-26b predicts breast cancer recurrence. Correlation between miR-26b expression and breast cancer recurrence was tested using Kaplan-Meier analyses of publicly-available miRNA expression array data from 152 ER positive, high-risk, primary breast tumors patients [52] mined using the Bioprofiling platform (http://www.bioprofiling.de/index.html).

**Supplementary Data S1.** Microarray data of miRNA expression levels from NFs and CAFs and normal and cancer epithelial cells from FFPE breast tissue. Microarray data of miRNA expression levels from the tissue culture model ‘NFs’ and ‘CAFs’.

**Supplementary Data S2.** MiRNAs consistently up- or down-regulated in CAFs compared with NFs in both FFPE breast tissue and the tissue culture model. Fold changes in both systems are shown for each miRNA along with the Geomean of the two values. Fold changes are not appropriate when miRNAs were undetectable in one of the matched samples; this is denoted by “n/a”.

**Supplementary Data S3.** Protein expression levels in triplicate samples of 26^k/d^ (T1, T2, and T3) and control^k/d^ (C1, C2, and C3) fibroblasts as determined by mass spectrometry. Significance of differential expression was determined by Student’s T Tests (p value) and by False Discovery Rates (FDR).
