# Supplementary material for: MiR-26b is down-regulated in carcinoma-associated fibroblasts from ER-positive breast cancers leading to enhanced cell migration and invasion
Source: J Pathol. 2013 Oct 9;231(3):388–99. doi: 10.1002/path.4248 (PMC4030585; doi:10.1002/path.4248)
Supplement: Table S1 — Pathological features of the breast cancer cases included in this study. [file path0231-0388-sd2.doc]

| **Usage** | **Type** | **Grade** | **LN status (+/-)** | **ER status (+/-)** | **Her2 status (+/-)** |
| --- | --- | --- | --- | --- | --- |
| Microarrays | ductal-NST | 2 | - | + | - |
|  |  |  |  |  |  |
| qPCR 1 | ductal-NST | 2 | - | + | - |
| qPCR 2 | ductal-NST | 2 | - | + | - |
| qPCR 3 | ductal-NST | 2 | - | + | - |
| qPCR 4 | ductal-NST | 2 | - | + | - |
| qPCR 5 | ductal-NST | 2 | - | + | - |
| qPCR 6 | ductal-NST | 2 | - | + | - |
| qPCR 7 | ductal-NST | 2 | - | + | - |
| qPCR 8 | ductal-NST | 2 | - | + | - |
| qPCR 9 | ductal-NST | 2 | - | + | - |
| qPCR 10 | ductal-NST | 2 | - | + | - |
| qPCR 11 | ductal-NST | 2 | - | + | - |
| qPCR 12 | ductal-NST | 2 | - | + | - |
| qPCR 13 | ductal-NST | 2 | - | + | - |
| qPCR 14 | ductal-NST | 2 | - | + | - |
|  |  |  |  |  |  |
| 1^o^ cultures 1 | ductal-NST | 2 | - | + | - |
| 1^o^ cultures 2 | ductal-NST | 3 | - | + | - |
| 1^o^ cultures 3 | ductal-NST | 1 | - | + | - |
| 1^o^ cultures 4 | Mixed | 1 | unknown | + | + |

**Table S1.** Pathological features of the breast cancer cases included in this study. NST: no special type; LN: lymph node; ER: estrogen receptor alpha.
