# Supplementary material for: MiR-26b is down-regulated in carcinoma-associated fibroblasts from ER-positive breast cancers leading to enhanced cell migration and invasion
Source: J Pathol. 2013 Oct 9;231(3):388–99. doi: 10.1002/path.4248 (PMC4030585; doi:10.1002/path.4248)
Supplement: Table S2 — Components of pathways identified by Gene Ontology analyses as enriched in the proteins differentially expressed between controlk/d and 26k/d fibroblasts. [file path0231-0388-sd3.doc]

|  | **Gene** | **Gene name** | **Fold Change** |
| --- | --- | --- | --- |
| **GROUP 1: cytoskeletal regulation by Rho GTPases** | | |  |
| 1 | ACTB | actin, beta | 2.58 |
| 2 | ACTG1 | actin, gamma 1 | 2.58 |
| 3 | ACTBL2 | actin, beta-like 2 | 4.39 |
| 4 | ARHGAP1 | Rho GTPase activating protein 1 | 2.53 |
| 5 | PAK1 | p21 protein (Cdc42/Rac)-activated kinase 1 | 1.52 |
| 6 | MYH10 | myosin, heavy chain 10, non-muscle | 5.45 |
| 7 | ARPC1A | actin related protein 2/3 complex, subunit 1A, 41kDa | down* |
| 8 | CFL2 | cofilin 2 (muscle) | 3.59 |
| 9 | CFL1 | cofilin 1 (non-muscle) | 1.69 |
| 10 | TUBB6 | tubulin, beta 6 class V | 2.91 |
| 11 | PFN1 | profilin 1 | 1.72 |
| 12 | ARPC5 | actin related protein 2/3 complex, subunit 5, 16kDa | down* |
| 13 | ROCK2 | Rho-associated, coiled-coil containing protein kinase 2 | down* |
| 14 | STMN1 | stathmin 1 | 1.69 |
|  |  |  |  |
| **GROUP 2: glycolysis/gluconeogenesis and TCA** | | |  |
| 1 | ALDOA | aldolase A, fructose-bisphosphate | 1.46 |
| 2 | SDHA | succinate dehydrogenase complex, subunit A, flavoprotein (Fp) | 2.68 |
| 3 | MDH1 | malate dehydrogenase 1, NAD (soluble) | 2.15 |
| 4 | PGK1 | phosphoglycerate kinase 1 | 2.02 |
| 5 | PGAM1 | phosphoglycerate mutase 1 (brain) | 2.06 |
| 6 | IDH1 | isocitrate dehydrogenase 1 (NADP+), soluble | 2.45 |
| 7 | DLST | dihydrolipoamide S-succinyltransferase (E2 component of 2-oxo-glutarate complex) | 1.92 |
| 8 | ENO1 | enolase 1, (alpha) | 1.84 |
| 9 | GAPDH | glyceraldehyde-3-phosphate dehydrogenase | 1.62 |

**Table S2.** Components of pathways identified by Gene Ontology analyses as enriched in the proteins differentially expressed between control^k/d^ and 26^k/d^ fibroblasts. Gene names and fold differences in expression are shown. Fold changes were calculated using mean expression levels in triplicate control^k/d^  samples and triplicate 26^k/d^ samples. *Fold changes are shown only when the protein was detected in both cell lines – when it was not detected in either line only the direction of deregulation is indicated.
