# Supplementary material for: MiR-26b is down-regulated in carcinoma-associated fibroblasts from ER-positive breast cancers leading to enhanced cell migration and invasion
Source: J Pathol. 2013 Oct 9;231(3):388–99. doi: 10.1002/path.4248 (PMC4030585; doi:10.1002/path.4248)
Supplement: Table S3 — Published miR-26b targets. [file path0231-0388-sd4.doc]

| **Target** | **Reference** |
| --- | --- |
| GATA4 | Han M, et al. (2012) GATA4 expression is primarily regulated via a miR-26b-dependent post-transcriptional mechanism during cardiac hypertrophy. Cardiovasc Res 93, 645-654 |
| PTEN | Palumbo T, et al. (2013) Functional screen analysis reveals miR-26b and miR-128 as central regulators of pituitary somatomammotrophic tumor growth through activation of the PTEN-AKT pathway. Oncogene 32, 1651-9 |
| ATF2 | Arora H, et al. (2011) Coordinated regulation of ATF2 by miR-26b in gamma-irradiated lung cancer cells. PLoS One 6, e23802 |
| ATM | Lin F, et al. (2012) miR-26b promotes granulosa cell apoptosis by targeting ATM during follicular atresia in porcine ovary. PLoS One 7, e38640 |
| CTDSP2 | Dill H, et al. (2012) Intronic miR-26b controls neuronal differentiation by repressing its host transcript, ctdsp2. Genes Dev 26, 25-30 |
| BDNF | Caputo V, et al. (2011) Brain derived neurotrophic factor (BDNF) expression is regulated by microRNAs miR-26a and miR-26b allele-specific binding. PLoS One 6, e28656 |
| COX2 | Ji Y, et al. (2010) MiRNA-26b regulates the expression of cyclooxygenase-2 in desferrioxamine-treated CNE cells. FEBS Lett 584, 961-967 |
| DNMT3b | Sandhu R, et al. (2012) Loss of post-transcriptional regulation of DNMT3b by microRNAs: a possible molecular mechanism for the hypermethylation defect observed in a subset of breast cancer cell lines. Int J Oncol 41, 721-732 |
| LEF1 | Zhang Z, et al. (2010) MicroRNAs regulate pituitary development, and microRNA 26b specifically targets lymphoid enhancer factor 1 (Lef-1), which modulates pituitary transcription factor 1 (Pit-1) expression. J Biol Chem. 285, 34718-34728 |
| EphA2 | Wu N, et al. Role of microRNA-26b in glioma development and its mediated regulation on EphA2. PLoS One 6, e16264 |
| SLC7A11 | Liu XX, et al. (2011) MicroRNA-26b is underexpressed in human breast cancer and induces cell apoptosis by targeting SLC7A11. FEBS Lett 585(9):1363-1367 |
| EZH2 | Koh CM, et al. (2011) Myc enforces overexpression of EZH2 in early prostatic neoplasia via transcriptional and post-transcriptional mechanisms. Oncotarget 2, 669-683 |
| cdc6  cyclin E1 | Zhu Y, et al. (2012) MicroRNA-26a/b and their host genes cooperate to inhibit the G1/S transition by activating the pRb protein. Nuc Acids Res 40, 4615-4625 |

**Table S3.** Published miR-26b targets.
