# Supplementary figures and images for: MiR-26b is down-regulated in carcinoma-associated fibroblasts from ER-positive breast cancers leading to enhanced cell migration and invasion
Source: J Pathol. 2013 Oct 9;231(3):388–99. doi: 10.1002/path.4248 (PMC4030585; doi:10.1002/path.4248)

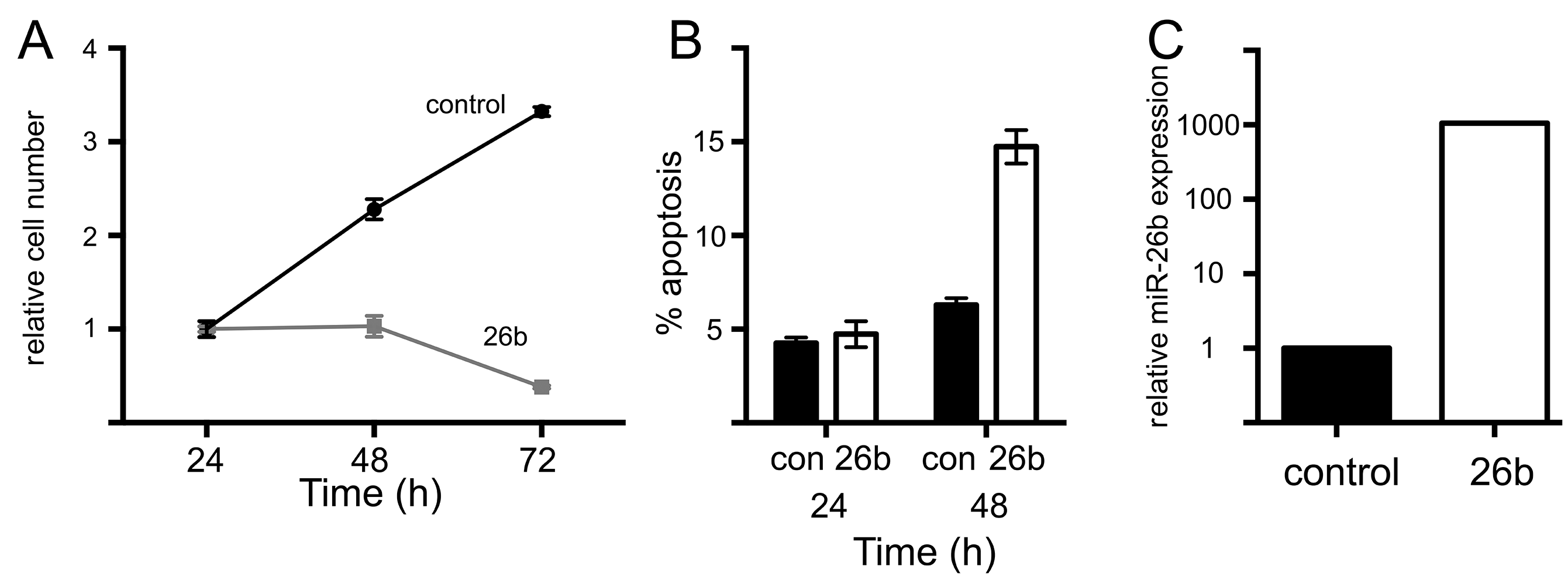

Supplement: Figure S1 — Transient miR-26b up-regulation in breast fibroblasts induced dramatic growth inhibition and apoptosis. [file path0231-0388-sd5.tif]

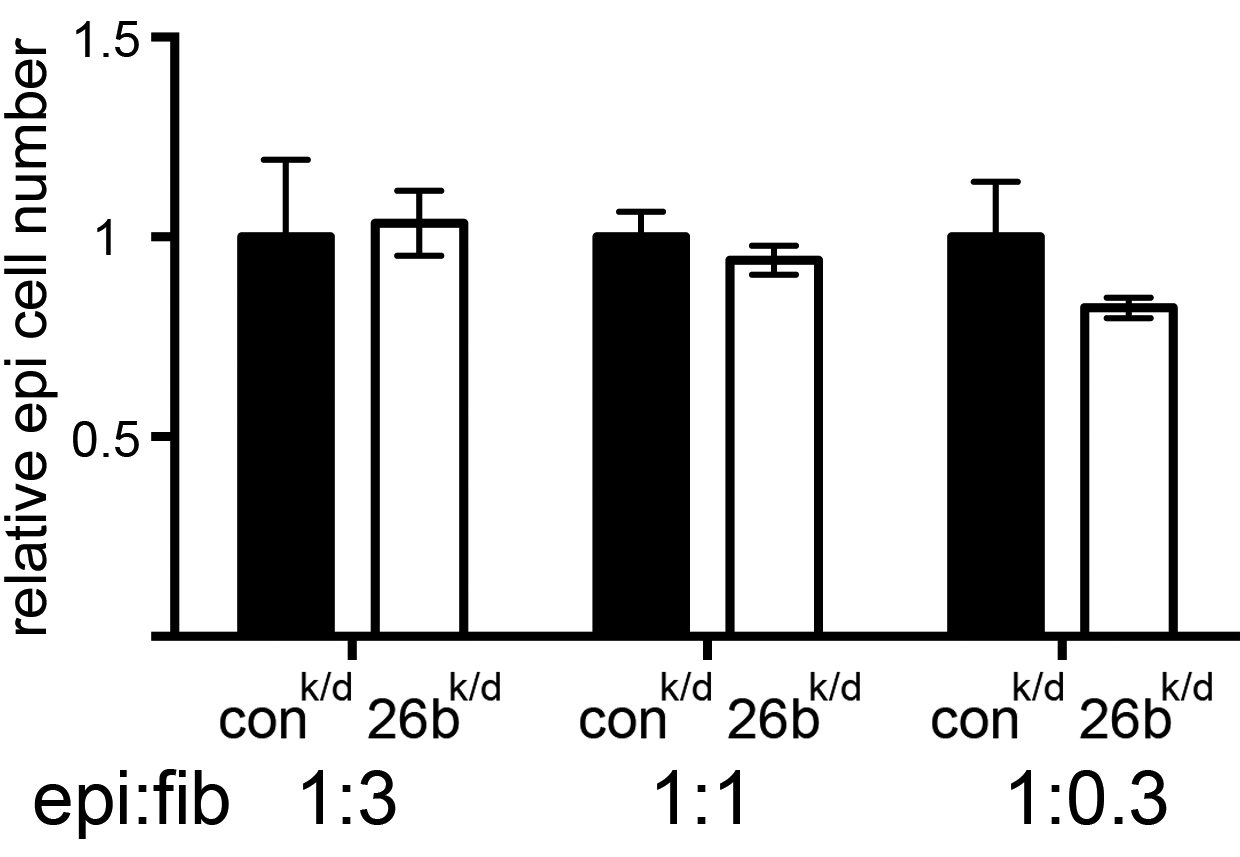

Supplement: Figure S2 — MiR-26b knock-down or control breast fibroblasts do not have differential influences on MCF7 cell growth at a range of different seeding densities. [file path0231-0388-sd6.tif]

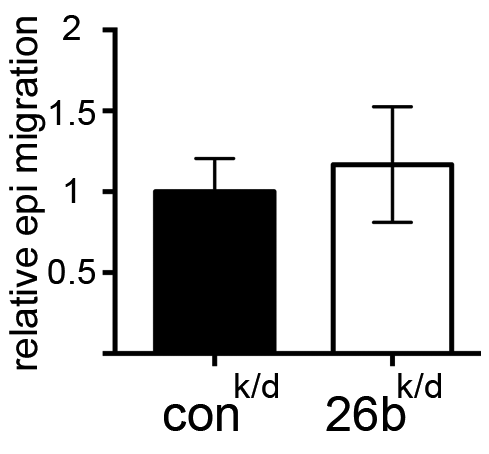

Supplement: Figure S3 — MCF7 cell migration is not stimulated by breast fibroblasts with reduced miR-26b when the cells are seeded in separate chambers of trans-wells. [file path0231-0388-sd7.tif]

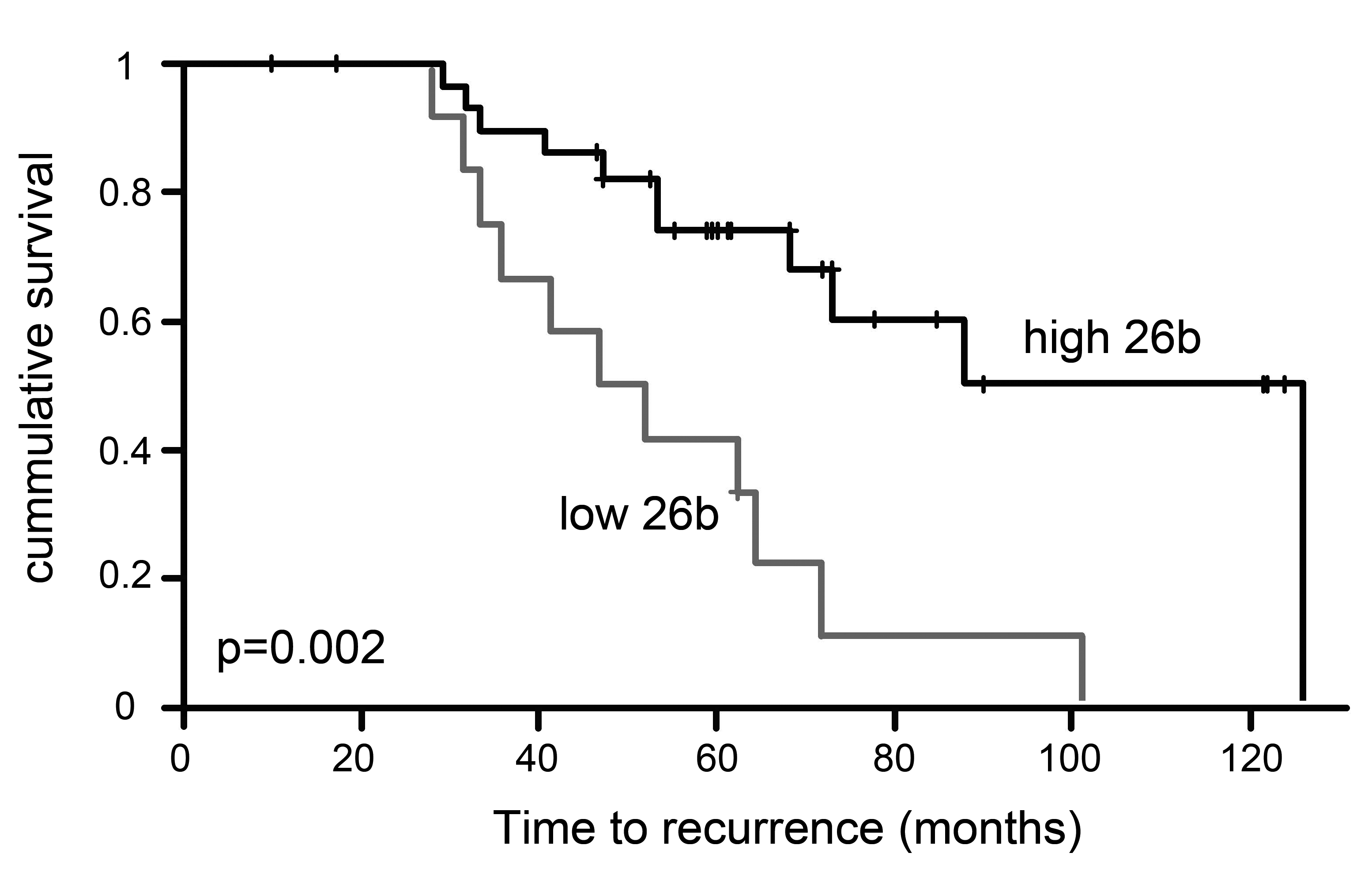

Supplement: Figure S4 — Expression of miR-26b predicts breast cancer recurrence. [file path0231-0388-sd8.tif]
